# A deep learning-based self-adapting ensemble method for segmentation in gynecological brachytherapy

**DOI:** 10.1186/s13014-022-02121-3

**Published:** 2022-09-05

**Authors:** Zhen Li, Qingyuan Zhu, Lihua Zhang, Xiaojing Yang, Zhaobin Li, Jie Fu

**Affiliations:** Shanghai Jiao Tong University Affiliated Sixth People’s Hospital, Xuhui District, Shanghai, China

**Keywords:** Deep learning, High-dose-rate brachytherapy, Auto-segmentation, Gynecological cancer

## Abstract

**Purpose:**

Fast and accurate outlining of the organs at risk (OARs) and high-risk clinical tumor volume (HRCTV) is especially important in high-dose-rate brachytherapy due to the highly time-intensive online treatment planning process and the high dose gradient around the HRCTV. This study aims to apply a self-configured ensemble method for fast and reproducible auto-segmentation of OARs and HRCTVs in gynecological cancer.

**Materials and methods:**

We applied nnU-Net (no new U-Net), an automatically adapted deep convolutional neural network based on U-Net, to segment the bladder, rectum and HRCTV on CT images in gynecological cancer. In nnU-Net, three architectures, including 2D U-Net, 3D U-Net and 3D-Cascade U-Net, were trained and finally ensembled. 207 cases were randomly chosen for training, and 30 for testing. Quantitative evaluation used well-established image segmentation metrics, including dice similarity coefficient (DSC), 95% Hausdorff distance (HD95%), and average surface distance (ASD). Qualitative analysis of automated segmentation results was performed visually by two radiation oncologists. The dosimetric evaluation was performed by comparing the dose-volume parameters of both predicted segmentation and human contouring.

**Results:**

nnU-Net obtained high qualitative and quantitative segmentation accuracy on the test dataset and performed better than previously reported methods in bladder and rectum segmentation. In quantitative evaluation, 3D-Cascade achieved the best performance in the bladder (DSC: 0.936 ± 0.051, HD95%: 3.503 ± 1.956, ASD: 0.944 ± 0.503), rectum (DSC: 0.831 ± 0.074, HD95%: 7.579 ± 5.857, ASD: 3.6 ± 3.485), and HRCTV (DSC: 0.836 ± 0.07, HD95%: 7.42 ± 5.023, ASD: 2.094 ± 1.311). According to the qualitative evaluation, over 76% of the test data set had no or minor visually detectable errors in segmentation.

**Conclusion:**

This work showed nnU-Net’s superiority in segmenting OARs and HRCTV in gynecological brachytherapy cases in our center, among which 3D-Cascade shows the highest accuracy in segmentation across different applicators and patient anatomy.

**Supplementary Information:**

The online version contains supplementary material available at 10.1186/s13014-022-02121-3.

## Introduction

The combination of external beam radiation therapy (EBRT) and HDR-BT is a standard care for treatment in gynecological cancers [1, 2], in which HDR-BT has proven to be indispensable and has a strong correlation with a higher survival rate [2–4].

In the HDR-BT treatment, contouring of OARs and HRCTV should be careful and accurate for better organ sparing and tumor control due to the high dose gradient in brachytherapy. However, unlike heaps of treatment planning time in EBRT, there is limited time for the planning procedure during HDR-BT because the radiation oncologists and medical physicists should finish the contouring in the shortest possible time to reduce the patient’s uncomfortableness and the possibilities of patient movement [5, 6]. It is estimated that a radiation oncologist needs 32 min on average to delineate the HRCTV and OARs for gynecologic malignancies [7]. The requirements of fast pace and accurate planning will put the entire workflow under high pressure, thus increasing planning errors. Moreover, the planner’s experience level and preferences would result in significant inter-and intra-observer variations [8, 9], further introducing more uncertainties in treatment planning and dose delivery [10–12].

Therefore, the contouring of OARs and HRCTV in HDR-BT is often considered the bottleneck in the clinical workflow [13, 14]. There is a strong need for a precise and fast automatic contouring tool in the clinic. For a long time, there have been attempts to automate the contouring process. Most of the studies focus on segmentation tools, including atlas-based and deep learning methods [15, 16]. In the past ten years, atlas-based auto-segmentation (ABAS) algorithms, which segment the contours based on a library of reference images, mapping elements to the target image using a deformable image registration algorithm, have been widely used for auto-segmentation. Kim et al. [17] segmented CTV and OARs in endometrial gynecological cancer and achieved the best dice of 0.75 as well as an average segmentation time of 45.1 s. Although ABAS increases the contouring efficiency, it still has some disadvantages. Kim stated organs isodense with their surroundings are not suitable subjects for ABAS. Teguh et al. [18] found ABAS does not perform well for small and thin OARs. Moreover, it is reported that approximately 5000 atlases should be included to achieve a segmentation level corresponding to clinical quality [19]. However, even for those studies using large databases, the atlas selection may be unreliable, potentially influencing the segmentation performance [20]. Finally, applicators and CT markers may bring metal artifacts to the CT images and degrade the image quality, which causes an undesirable effect on the segmentation [21]. Thus, the ABAS method does not have many clinical applications because this approach is limited in accuracy, thereby leading to slight improvement at best, in contouring efficiency.

With the increasing popularity of deep learning, multiple architectures have been developed and applied in medical image segmentation, such as Cascaded U-Net [22–24], VGGNet [25], AlexNet [26, 27], DenseNet [28, 29], ResNet [30, 31], some of these methods have achieved good results and outperformed the ABAS for the majority of clinical cases [32–35]. Despite the good performance achieved by these networks, their applicability to specific image segmentation is often limited. The task-specific design and configuration of a network require careful fine-tuning. Slight variations in hyperparameters could lead to significant differences in performance. A fine-tuned neural network model for one specific task is highly possible to fail in other application scenarios [36].

nnU-Net is the first fully automatic framework for biomedical segmentation [37]. It consists of 2D, 3D and 3D Cascade U-Net based on several convolution and deconvolution layers, with skip connections [38]. The most attractive part of nn U-net is the automatic configuration of the pre-and post-processing, network architecture, and training for any new task. This robust strategy even outperforms highly specialized solutions on 23 public datasets used in international biomedical segmentation competitions [37]. Similar standardized schemes based on self-adapted architecture have not been applied in gynecological cancer and HDR brachytherapy treatment. In this work, nnU-Net is proposed for gynecological cancer patients in HDR-BT. It has proved to have better segmentation accuracy than existing methods and can be easily translated to clinical practice.

## Methods and materials

### Patient selection and contouring

62 Patients were included in the retrospective study approved by the institutional review board. Each patient contains 2–6 fractions; and each fraction has a unique CT structure set. A total of 237 cases were included in this study. 207 cases were used for training and 30 cases for testing. A “case” in this context indicates one single fraction in the treatment. These patients were randomly selected from the gynecological patients between January 2019 and September 2021. All the CT images were acquired using 120 kV and 60mAs at a GE 128 slice CT (Discovery, GE Healthcare, Inc.). The slice thickness and slice increment were 2.5*2.5 mm; and the image resolution was 512*512. The average pixel spacing in axial image is 0.75 mm*0.75 mm. All the scans used same image acquisition and reconstruction protocol. Each patient was treated with an applicator set among Tandem and Ovoid applicator (T + O), Vaginal Multi-Channel applicator, Ovoid applicator, free needles, and a tandem applicator with up to 10 interstitial needles (T + N) (see Additional file 1: Table S1). The HRCTV, rectum and bladder were manually delineated using the Oncentra System (Elekta, Stockholm, Sweden) by an experienced radiation oncologist. All the contours were reviewed and edited by another more experienced radiation oncologist. The results confirmed by the second oncologist were considered the final delineations (i.e., the ground truth) for training and testing.

### Geometric evaluation

#### Quantitative evaluation

To evaluate the auto-segmentation performance, we compared the predicted segmentation generated by the models with the provided ground truth. We used the dice similarity coefficient (DSC) [39], average surface distance (ASD), and 95% Hausdorff distance (HD95%) [40] as three indicators to evaluate the accuracy of segmentation. These indicators are the most widely used metrics for quantitatively assessing segmentation quality in auto-segmentation.

#### Qualitative evaluation

Two radiation oncologists (5-year and 20-year clinical experience) evaluated the auto-segmentation results in test set visually and graded the results using a 4-point Likert scale [41], in which Point 1 indicates no visible segmentation errors; Point 2 indicates minor segmentation errors; Point 3 indicates major segmentation errors; Point 4 indicates failed segmentation/no segmentation.

### Dosimetric evaluation

The dosimetric evaluation was performed to illustrate the difference in OARs and HRCTV between predicted segmentation and human contouring. Standard deviation over the residuals was considered as a measure of model error. The prescription dose was 5-6 Gy in each fraction and each patient contained 2–6 fractions. Plans were created considering the external beam and BT equivalent dose in 2 Gy fractions (EQD2). The OAR dose constraints and the prescription dose were based on American Brachytherapy Society HDR-BT guidelines for locally advanced gynecological cancer [42] and later updated EMBRACE-II trial [43]. For HRCTV, D90% (the minimum dose given to 90% of the target volume), and V100%, V150%, V200% (the target volume enveloped by 100%, 150%, and 200% of the prescribed dose) were evaluated. For OARs, the minimum dose received by 2cm3, 1cm3, 0.1cm3 (D2cc, D1cc, D0.1cc), and the maximum dose (D_max_), were evaluated. Since the dose distribution, and by extension, dose-volume parameters, can vary largely between different plans, a customized python program was developed to calculate the dose-volume parameters based on predicted contours (P_predicted_) using the dose map of original plan created based on manual contours (P_orginal_). Namely, the P_predicted_ and P_orginal_ shared the same dose map, simulating the same applicator position, source dwell position and dwell time. Model performance was quantified by calculating the residual of dose-volume parameters between P_predicted_ and P_orginal_.

### Auto-segmentation network

In this study, nnU-Net was selected to provide a standardized workflow to achieve accurate and reproducible segmentation. The program was implemented with Python 3.7, and performed on a workstation platform with an NVIDIA GeForce RTX 3060 GPU in an Ubuntu 20.04.3 operating system.

#### Network architecture and training workflow

**Fig. 1:**
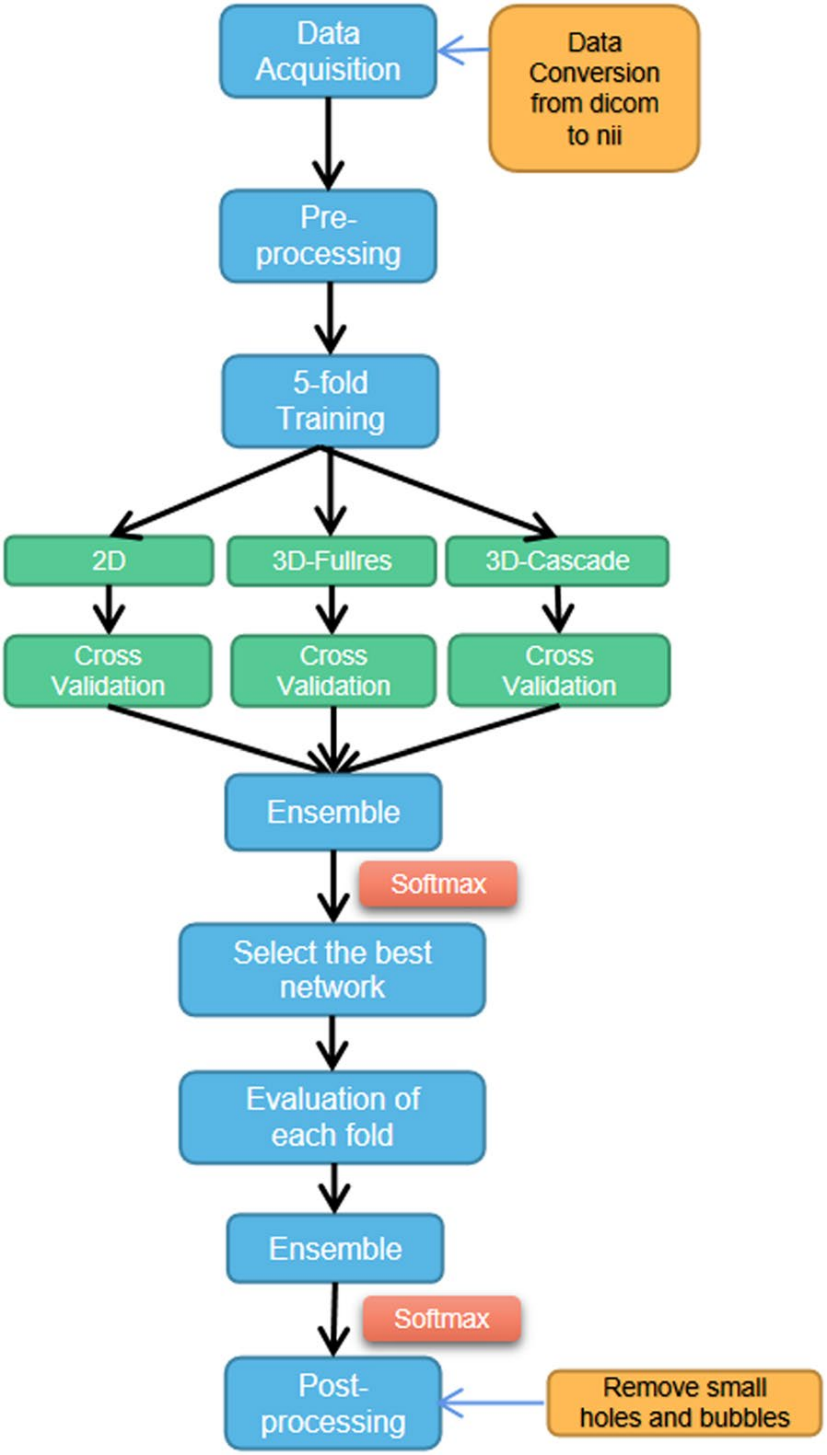
An overview of training workflow

The architecture template of nnU-net is a ‘U-Net-like’ encoder-decoder with skip connections and instance normalization. It provides three architectures based on the U-Net backbone: a two-dimensional (2D) U-Net, a three-dimensional (3D) U-Net training all images at full image resolution (3D-Fullres), and a 3D U-Net cascade network (3D-Cascade). The 3D-Cascade network contains two U-Nets, the first 3D U-Net creates coarse segmentation maps on down-sampled images (3D-Lowres); and the second 3D U-Net operates on full resolution images to refine the segmentation map created by the first one. An overview of the training workflow is shown in Fig. 1. In data acquisition, all the data was converted to nii format. Then, the data was prepossessed using data augmentation, which includes scaling, rotation, adding Gaussian blur and Gaussian noise, simulating low-resolution gamma mirroring, and Gamma augmentation. In training phase, fivefold training was used for each architecture. In ensemble, nnU-Net empirically chooses the best model (or combination of two) from 2D U-Net, 3D-Fullres or 3D-Cascade according to the five-fold cross-validation results. Ensemble is processed by averaging softmax probabilities. After training, the post-processing is triggered for individual classes by removing all small holes inside the OARs and HRCTV.

#### Hyperparameter setting

In the training period, each architecture went through five-fold cross-validation and each fold ran for 1000 epochs with an epoch size of 250. The optimizer used stochastic gradient descent with a high initial learning rate (0.01) and a large momentum (μ = 0.99). We adapted ‘poly’ learning rate decay strategy (1−epoch/epochmax)0.9 to accelerate convergence. The activate function was leaky ReLU. To improve the training stability and segmentation accuracy, nnU-Net used a combination of dice and cross-entropy as loss function empirically. The batch and patch sizes are shown in Table 1. Each network's batch size and patch size were adjusted according to the image size and GPU’s computing power. To ensure robust optimization, the batch size is set to a minimum of 2 and is increased until GPU memory is maxed out. A large patch size could provide additional background information to help the network make decisions. As a result, if GPU is allowed, we maximize the patch size under the condition of a batch size of 2.

**Table 1:** Detailed information of input images before training

	2D	3D-fullres	3D-lowres
Median image size	512 × 512	63 × 512 × 512	63 × 354 × 354
Median target spacing	0.75 × 0.75	2.5 × 0.75 × 0.75	2.5 × 1.0838 × 1.0838
Patch size	512 × 512	28 × 256 × 256	40 × 224 × 224
Batch size	12	2	2

### Statistical analysis

We considered possible variables that could potentially explain the variance of auto-segmentation quality through ANOVA (analysis of variance). *p* Values less than 0.05 were considered significant after Bonferroni correction. The independent variables are applicator type, organ type, and tumor location (vagina, uterus, or both). The dependent variables are DSC values in geometric evaluation. Moreover, Cohen’s kappa (κ) evaluated the inter-observer agreement between the two radiation oncologists at qualitative evaluation. To test for significant differences (*p* < 0.05) between the three architectures’ performance, we used an independent two-sample *t*-test as calculated with SciPy for OARs and HRCTV.

## Results

### Geometric evaluation

#### Quantitative evaluation

All three architectures successfully segmented the bladder, rectum and HRCTV on the test dataset. The performance for each metric is shown in Table 2. A general trend in the test dataset showed that the DSC in order from highest to lowest was the bladder, rectum and HRCTV, and DSC values in rectum and HRCTV were almost identical. A similar trend was observed in the ASD and HD95%, in which bladder achieved the highest performance and rectum had a comparable performance with HRCTV.

**Table 2:** Auto-segmentation network performance compared to manual segmentation (i.e., ground truth) on bladder, rectum, and HRCTV for each metric

	Model	DSC	HD95%	ASD
Bladder	2D	0.917 ± 0.054	4.381 ± 2.5	1.372 ± 1.073
	3D-fullres	0.935 ± 0.05	3.495 ± 2.291	0.95 ± 0.56
	3D-cascade	0.936 ± 0.051	3.503 ± 1.956	0.944 ± 0.503
	Ensemble	0.935 ± 0.05	3.495 ± 2.291	0.95 ± 0.56
Rectum	2D	0.808 ± 0.106	9.97 ± 8.267	3.949 ± 4.178
	3D-fullres	0.816 ± 0.098	8.137 ± 7.581	3.719 ± 3.084
	3D-CASCADE	0.831 ± 0.074	7.579 ± 5.857	3.6 ± 3.485
	Ensemble	0.831 ± 0.074	7.579 ± 5.857	3.6 ± 3.485
HRCTV	2D	0.763 ± 0.136	9.186 ± 5.347	2.718 ± 1.631
	3D-fullres	0.806 ± 0.108	8.815 ± 6.485	2.46 ± 1.756
	3D-cascade	0.836 ± 0.07	7.42 ± 5.023	2.094 ± 1.311
	Ensemble	0.806 ± 0.108	8.815 ± 6.485	2.46 ± 1.756

The highest DSC, of any network, in the evaluation dataset as compared to manual segmentations (i.e., ground truth) for each contouring were 0.936 ± 0.051 (bladder in 3D-Cascade), 0.831 ± 0.074 (rectum in 3D-Cascade), and 0.836 ± 0.07 (HRCTV in 3D-Cascade). The lowest HD95%, of any network, were 3.495 ± 2.291 (bladder in 3D-Fullres), 7.579 ± 5.857 (rectum in 3D-Cascade), and 7.42 ± 5.023 (HRCTV in 3D-Cascade). The lowest ASD, of any network, were 0.944 ± 0.503 (bladder in 3D-Cascade), 3.6 ± 3.485 (rectum in 3D-Cascade), and 2.094 ± 1.311 (HRCTV in 3D-Cascade). Ensemble has the same geometric results with 3D-Fullres for bladder and HRCTV, as well as 3D-Cascade for rectum.

#### Network architecture comparison

**Fig. 2:**
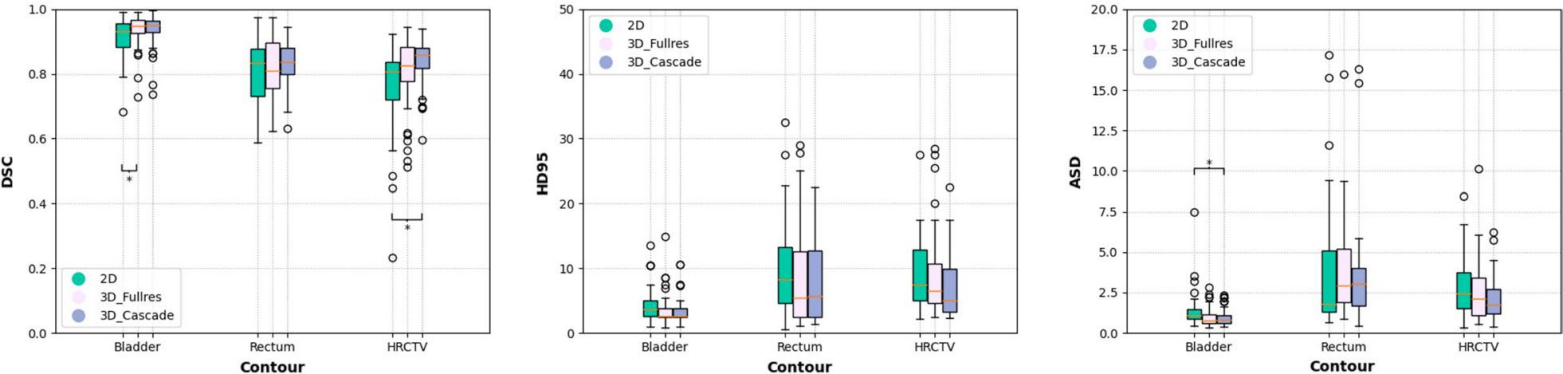
Comparison of the auto-contouring performance of three network architectures, as assessed with DSC, ASD, and HD95%. Significant differences between 2D, 3D-Fullres, and 3D-Cascade are marked with an asterisk *p < 0.05

**Fig. 3:**
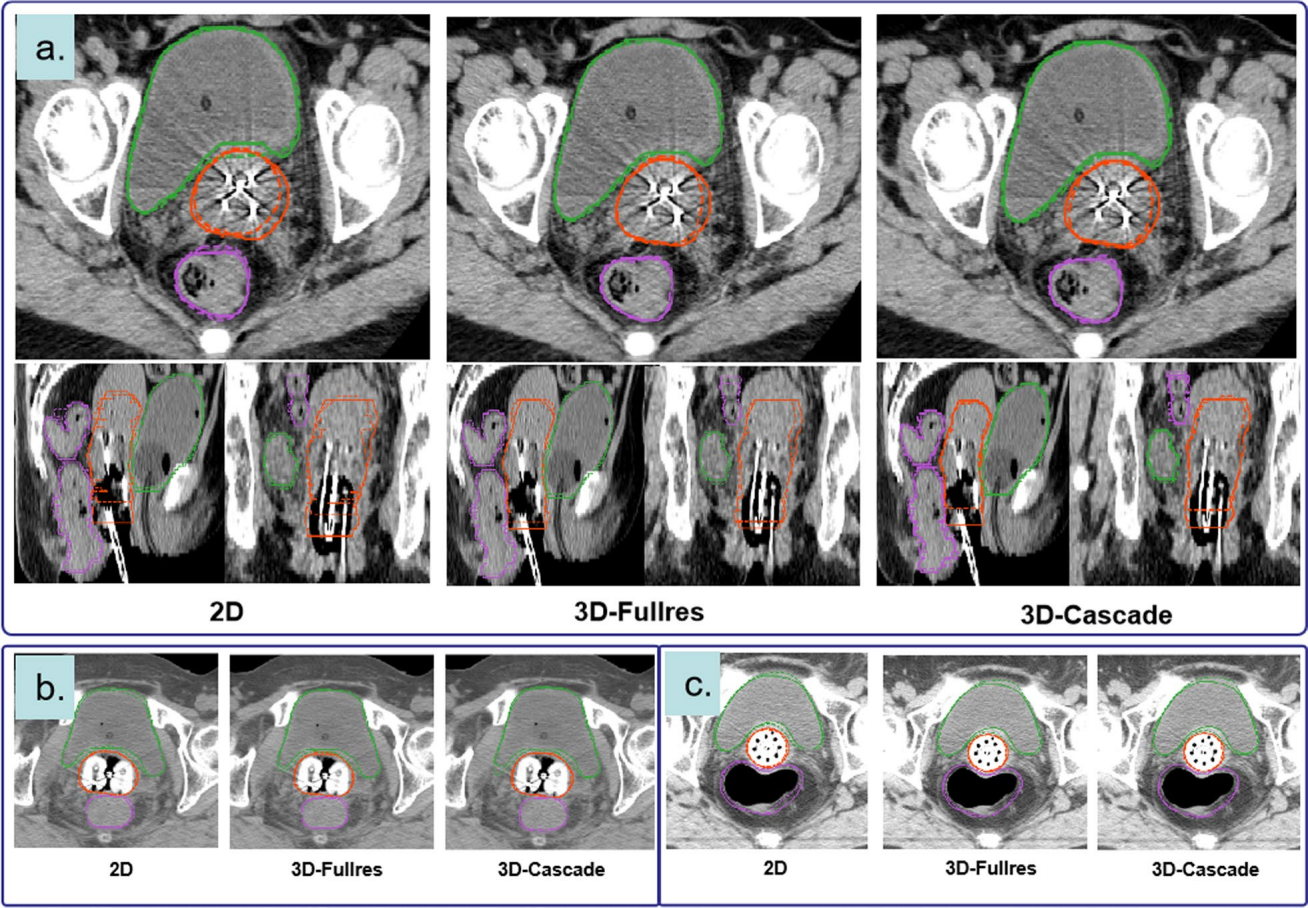
Visualization of segmentation in axial, sagittal, and coronal views with manual contouring (solid line) and auto-segmentation (dashed line): rectum (purple), bladder (green), and HRCTV (orange). All three architectures have a god segmentation in cervical cases inserted with different applicators (**a** Needles+Tandem Applicator, **b** Ovoid Applicator, **c** Vaginal Multi-channel Applicator)

Figure 2 shows a comparison of auto-segmentation performance for the 2D, 3D-Fullres, and 3D-Cascade. In general, 3D networks perform better than 2D for all evaluation metrics. The addition of low-resolution network in 3D-Cascade has relatively improved performance compared with 3D-Fullres, with slightly higher DSC, lower ASD, and HD 95%. The auto-segmentation results of each network architecture are shown in Fig. 3. All three networks have a good segmentation for OARs and HRCTV.

#### Qualitative evaluation

**Fig. 4:**
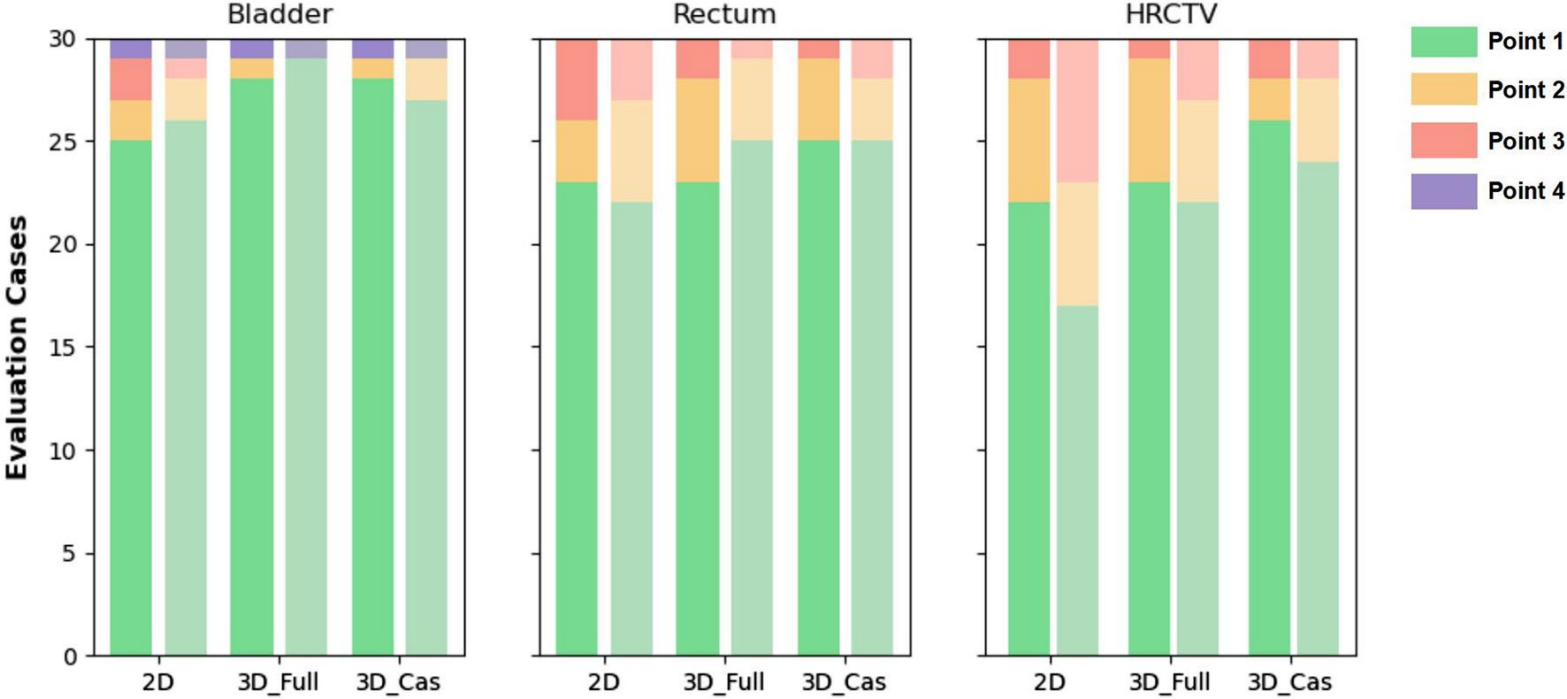
Two radiation oncologists evaluated qualitative segmentation results. A stacked bar chart demonstrates the distribution of qualitative evaluation scores (Point 1–Point 4) of three network results. The qualitative results of first and second radiation oncologists are shown in dark and light, respectively. Most segmentations showed no error (Point 1). Single cases showed only minor (Point 2) errors. Only one case showed failed segmentation due to contrast enhanced agent in the bladder (Point 4)

In general, qualitative evaluation of the segmentation performance revealed high accuracy for all the OARs and HRCTV in 2D, 3D-Fullres, and 3D-Cascade networks. Most of the data has achieved point 1, indicating that the predicted segmentation is visually flawless and clinically acceptable. At least an average of 65% (HRCTV in 2D network) of the evaluation data had no obvious segmentation errors, achieving point 1 in this study. Errors of the second level (point 2), indicating minor segmentation errors observed in several slices, were observed at the top slice in rectum (see Additional file 1: Fig. S1a, b). Compared with bladder and rectum, HRCTV segmentations showed a marginally higher rate of minor errors (point 2). The third level (point 3, major segmentation errors observed in most slices) were noticed only in a few single cases in which abnormal anatomy (e.g., large air bubbles in the bladder) exists (see Additional file 1: Fig. S1c). Failed segmentation (point 4, the object was not segmented) only occurred in one case because the contrast-enhanced agent resided in the bladder (see Additional file 1: Fig. S1d). Overall, the bladder segmentation showed the best qualitative results compared with the rectum and HRCTV. Figure 4 demonstrates the qualitative segmentation results. Good interobserver agreement was achieved on 2D (κ = 0.67), 3D-Fullres (κ = 0.69), and 3D-Cascade (κ = 0.78).

### Dosimetric evaluation

To evaluate the dosimetric accuracy, we compared the dose-volume parameters obtained from predicted contours with manually delineated contours (Table 3). The prescription dose given to the patients were 6 Gy (15 cases), 5.5 Gy (6 cases) and 5 Gy (9 cases). The average difference for △D90% in HRCTV is 0.46±1.2, 0.43±0.34, and 0.21±0.53 for the prescription dose of 6 Gy, 5.5 Gy, 5 Gy, respectively. For OARs, the average difference in D2cc is smaller than 15%.

**Table 3:** Results of dosimetric parameters for bladder, rectum and HRCTV

	HRCTV				Bladder				Rectum		
	Rx = 6	Rx = 5.5	Rx = 5		Rx = 6	Rx = 5.5	Rx = 5		Rx = 6	Rx = 5.5	Rx = 5
D_90%_	0.46 ± 1.2	0.43 ± 0.34	0.21 ± 0.53	D_2cc_	0.88 ± 0.67	0.82 ± 0.06	0.23 ± 0.13	D_2cc_	0.66 ± 0.64	0.59 ± 0.38	0.32 ± 0.25
V_100%_	3.28 ± 4.22	3.22 ± 2.37	9.37 ± 13.12	D_1cc_	0.97 ± 0.72	0.93 ± 0.08	0.21 ± 0.02	D_1cc_	0.72 ± 0.69	0.66 ± 0.48	0.37 ± 0.26
V_150%_	1.76 ± 2.42	1.81 ± 1.67	5.96 ± 10.25	D_0.1cc_	1.22 ± 0.98	1.06 ± 0.08	0.18 ± 0.19	D_0.1cc_	0.86 ± 0.96	0.52 ± 0.8	0.41 ± 0.23
V_200%_	0.99 ± 1.47	1.14 ± 0.98	3.85 ± 8.03	D_max_	1.31 ± 1.29	1.2 ± 0.3	0.1 ± 0.23	D_max_	0.95 ± 1.5	0.42 ± 1.06	0.29 ± 0.24

All values are described in the form of mean ± standard deviation

*Rx is the prescription dose. The unit is Gy for D_90%_, D_2cc_, D_1cc_, D_0.1cc_, and D_max_, and cc for V_100%_, V_150%_, and V_200%_

### Statistical analysis results

Comparison of three network architectures' auto-contouring performance as evaluated by a DSC, HD, and ASD. In general, 3D networks showed significant improvements over the 2D network. Statistical differences were found in the bladder (2D and 3D-Fullres in DSC, 2D and 3D-Cascade in ASD) and HRCTV (2D and 3D-Cascade in DSC). The detailed p-values are shown in Additional file 1: Table S2.

Analysis of variance after Bonferroni correction demonstrated that the applicator type and the organ type were statistically significant factors affecting segmentation results. DSC was statistically significantly better in HRCTV segmentation for Vaginal Multi-Channel applicator and Ovoids applicator compared with T + N, T + O, and free needles (*p* < 0.05), and for the bladder compared with rectum and HRCTV (*p* < 0.05). Tumor location had no statistically significant effect on the segmentation results.

## Discussion

Auto-segmentation is highly desired in brachytherapy treatment planning since patients can hardly hold on to one position for a long time. Moreover, minimizing the HRCTV and OARs contouring variability can improve plan quality consistency, thus allowing dose-escalation strategy in HRCTV. Currently, some commercial systems have been applied and evaluated in clinic to test their accuracy, which would greatly benefit the clinical workflow. Chen et al. reported a whole-body net (Deep Voxel. Inc.). They tested its accuracy and efficiency in delineating all major OARs in the entire body and achieved average DSCs of 0.84 and 0.81 [44]. Guo et al. conducted the dosimetric of OARs between their in-house and a learning-based commercial auto-segmentation system (United Imaging Healthcare) with manual contouring. They found no significant difference for most cases in PTV and OAR doses [45]. In this study, we employed nn U-net, a self-adapting ensemble method comparable to a commercial system, for simultaneous multi-organ contouring in gynecological brachytherapy. Another key benefit of nnUnet is that it provides a standardized workflow without laborious fine-tuning, making it simple to deploy and potentially widespread in the clinic.

The nnU-net method has three architectures followed by an ensemble method to select the best architecture for each task. The ensemble would automatically pick the best performing method (or ensemble of methods) as the final model for the test. In our study, the test results showed that nnUnet picked the best architecture instead of the ensemble results after comparing the results between single architecture and ensemble results. In general, 3D architecture outperformed 2D slightly and reached competitive quantitative performance with DSC values well above 0.8. The performances of deep learning-based auto-contouring in gynecological cancer from other published papers are shown in Table 4. Two brachytherapy studies and two external-beam radiation therapy studies were included for reference. Compared with previous studies, our method has the highest performance in bladder and rectum segmentation, concerning a DSC of 0.936 ± 0.051 for bladder and 0.831 ± 0.074 for the rectum. The DSC of automated HRCTV segmentation (0.836 ± 0.07) was slightly inferior compared with the EBRT study [46] (0.86 ± 0.02) and Rhee’s BT study (0.86 ± 0.08) [47]. The complex shape can explain this and different applicators used in BT compared with EBRT cases and the large data set used in Rhee’s BT study. Overall, as far as directly comparable, the observed DSC value of automated segmentation in our study was competitive compared with similar previous research. The possible reason may be the larger training data set in this study (more than 200 training cases), which has more anatomical variability and applicator types than similar studies using a small dataset.

**Table 4:** Summary of deep learning-based auto-segmentation results in gynecological cancer from other groups

Publication	Data type	Training cases	Testing cases	Method	Organ	DSC
Zhang et al. [48]	BT	73	18	DSD-UNET	Bladder	0.869 ± 0.032
					Rectum	0.821 ± 0.05
					HRCTV	0.829 ± 0.041
				3D-UNET	Bladder	0.802 ± 0.041
					Rectum	0.771 ± 0.062
					HRCTV	0.742 ± 0.062
Wang et al. [46]	EBRT	100	25	3D-CNN	Bladder	0.91 ± 0.06
					Rectum	0.81 ± 0.04
					HRCTV	0.86 ± 0.02
Liu et al. [49]	EBRT	77	14	Improved UNET	Bladder	0.924 ± 0.046
					Rectum	0.791 ± 0.032
Rhee et al. [47]	BT	2254	140	CNN	Bladder	0.89 ± 0.09
					Rectum	0.81 ± 0.09
					HRCTV	0.86 ± 0.08
Our method	BT	205	30	nnU-NET	Bladder	0.936 ± 0.051
					Rectum	0.831 ± 0.074
					HRCTV	0.836 ± 0.07

If multiple network architectures are reported in the literature, the best-performing result was selected. The highest performance results (3D-Cascade) in our study were used for comparison. DSD-UNET: 3D-UNET incorporating residual connection, dilated convolution, and deep supervision

Generally, bladder has the best performance with an average DSC of 0.936 ± 0.051. The reason could be the significantly different CT values in the bladder compared with other organs in the pelvis. The architecture did not significantly differ between 2 and 3D in the bladder and rectum; the main reason for this could be the non-progressive change between different slices, especially in the upper part of the rectum and the lower part of the bladder. According to our ANOVA test, the factors affecting the segmentation accuracy include the applicator and organ types. HRCTV contouring has higher performance in the vaginal applicator and ovoid applicator. The possible reason could be fewer metal artifact in the vaginal and ovoid applicator. In addition, the segmentation accuracy did not significantly differ among different tumor locations.

Based on these results, we feel it possible to integrate these trained models in clinical workflow under staff supervision to solve the tricky problems in gynecological brachytherapy. However, there are still some limitations before implementation. Firstly, nnUnet is set to be trained and tested on an Ubuntu system, which may limit the application, especially for those users unfamiliar with Linux. We are now writing some patches to make it also compatible with Windows operating system. Secondly, the training process is quite time-consuming and requires a large amount of GPU resources. Thirdly, no cross-validation strategy was used to test performance on the testing data set (the aforementioned 5-fold validation was only used in training). This reduces the trustability of the performance measures, the robustness of the model and the reproducibility of the result. More clinical validation tests are appreciated to evaluate the model’s robustness in the future. In our study, we trained these models at an NVIDIA GeForce RTX 3060 GPU setting and we spent around 20 h for each fold in 2D and 65 h in 3D (see Additional file 1: Table S3). We plan to improve and simplify the training process in the future by reducing the number of training epochs or optimizing the network design to save time and improve training efficiency. Thirdly, the total time required for prediction for all OARs and HRCTV is relatively long, taking on an average of 2.7 min (2D), 14 min (3D-Fullres network), 17.5 min (3D-Cascade network), and 14.8 min (Ensemble) at an NVIDIA GeForce RTX 3060 GPU setting (Table 5). 3D-Cascade has the longest prediction time because it contains two U-Nets, and the prediction time increases rapidly with network architecture complexity. Moreover, the ensemble prediction time for bladder and HRCTV is similar with 3D-Fullres, and for rectum is similar to 3D-Cascade; the reason could be the ensemble empirically selected 3D-Fullres/3D-Cascade as the training model for testing. Since the prediction time for 5 folds is relatively long, we also calculate the prediction time in a single fold (fold 0) and compare it with the prediction time of all five folds. Ensemble has no fold0 prediction time because the contours in ensemble should be generated using all the five-fold images aggregated by softmax. The prediction time for one single fold vastly decreased to one-fifth of the five folds. Using the well-trained one-fold model in the clinic can improve the prediction efficiency. As shown in Table 6, the corresponding prediction accuracy of one-fold is slightly worse than five folds. This requires clinical users to make trade-offs between predicting accuracy and efficiency. Another possible solution could be using a more powerful graphic unit to increase the calculation speed. Further work related to architecture improvement or compression to accelerate the prediction speed is also a good research orientation.

**Table 5:** Time efficiency of different networks

Time (s)	2D	2D/fold0	3D-fullres	3D-fullres/fold0	3D-cascade	3D-cascade/fold0	Ensemble
Bladder	53.5	13.4	130.2	30.9	149.5	40.2	130.6
Rectum	54.8	13.7	256.4	55.5	278.9	65.8	278.9
HRCTV	57.4	14.3	476.7	97.1	623.2	131.9	479.1
Total	165.7	41.4	863.3	183.5	1051.6	237.9	888.6

Fold 0 is the first fold in each network

**Table 6:** DSC values for the first fold (fold0) and 5 folds

	Model	DSC-fold0	DSC-5folds
Bladder	2D	0.902 ± 0.084	0.917 ± 0.054
	3D-fullres	0.917 ± 0.231	0.935 ± 0.05
	3D-cascade	0.908 ± 0.045	0.936 ± 0.051
	Ensemble	–	0.935 ± 0.05
Rectum	2D	0.795 ± 0.115	0.808 ± 0.106
	3D-fullres	0.805 ± 0.152	0.816 ± 0.098
	3D-cascade	0.820 ± 0.131	0.831 ± 0.074
	Ensemble	–	0.831 ± 0.074
HRCTV	2D	0.741 ± 0.112	0.763 ± 0.136
	3D-fullres	0.780 ± 0.091	0.806 ± 0.108
	3D-cascade	0.813 ± 0.102	0.836 ± 0.07
	Ensemble	–	0.806 ± 0.108

In deep learning-based image segmentation area, lots of novel architectures are proposed in organ segmentation. However, fine-tuning of the hyper-parameters is tedious and time-consuming. Moreover, the generalizability and feasibility of clinical application needs further validation. In this study, we use nn U-net, a self-configuring and fully automated framework with a robust training strategy for segmentation. It systematizes the complex process of manual configuration instead of proposing a new network architecture, loss function or training scheme and achieved fairly good results [37]. In the future, we are going to extend the application of nn U-net to other medical image segmentation areas.

## Conclusion

In this work, we have shown that it is feasible to use a standardized nnU-net method for OARs and HRCTV segmentation in gynecological cancer. In our cases, the results show that combining a low-resolution and high-resolution U-net (3D-Cascade) has the highest accuracy in segmentation. With this 3D-Cascade network, high segmentation accuracy was obtained across different applicators and patient anatomy. Such performance would be beneficial to the clinical workflow by reducing the interobserver variations, releasing radiation oncologists’ and physicists’ burden, reducing patients’ pain, and increasing the planning efficiency in gynecological cancer treatment to a large extent.

## Supplementary Information

**Additional file 1.** Supplemental material.

## Data Availability

All data generated or analyzed during this study are included in this published article [and its supplementary information files]. Programming code of nnUnet: https://github.com/MIC-DKFZ/nnUNet.

## Abbreviations

OAR: Organs at risk
HRCTV: High-risk clinical tumor volume
HDR-BT: High-dose-rate brachytherapy
DSC: Dice similarity coefficient
HD95%: 95% Hausdorff distance
ASD: Average surface distance
EBRT: External beam radiation therapy
ABAS: Atlas-based auto-segmentation
T + O: Tandem and ovoid applicator
T + N: Tandem applicator with up to 10 interstitial needles
ANOVA: Analysis of variance
