# Supplementary material for: A deep learning-based self-adapting ensemble method for segmentation in gynecological brachytherapy
Source: Radiat Oncol. 2022 Sep 5;17:152. doi: 10.1186/s13014-022-02121-3 (PMC9446699; doi:10.1186/s13014-022-02121-3)
Supplement: Supplementary file 1 — Additional file 1. Supplemental material. [file 13014_2022_2121_MOESM1_ESM.docx]

Supplemental Material

|  | Train | Test | Total Number |
| --- | --- | --- | --- |
| Tandem + Ovoid | 33 | 5 | 38 |
| Ovoid Only | 51 | 7 | 58 |
| Tandem + Needles | 48 | 7 | 55 |
| Needles Only | 37 | 5 | 42 |
| Multi Channel Vaginal | 38 | 6 | 44 |
| Total Fraction/Case Number | 207 | 30 |  |

Table 1. Number of cases used for training and testing.


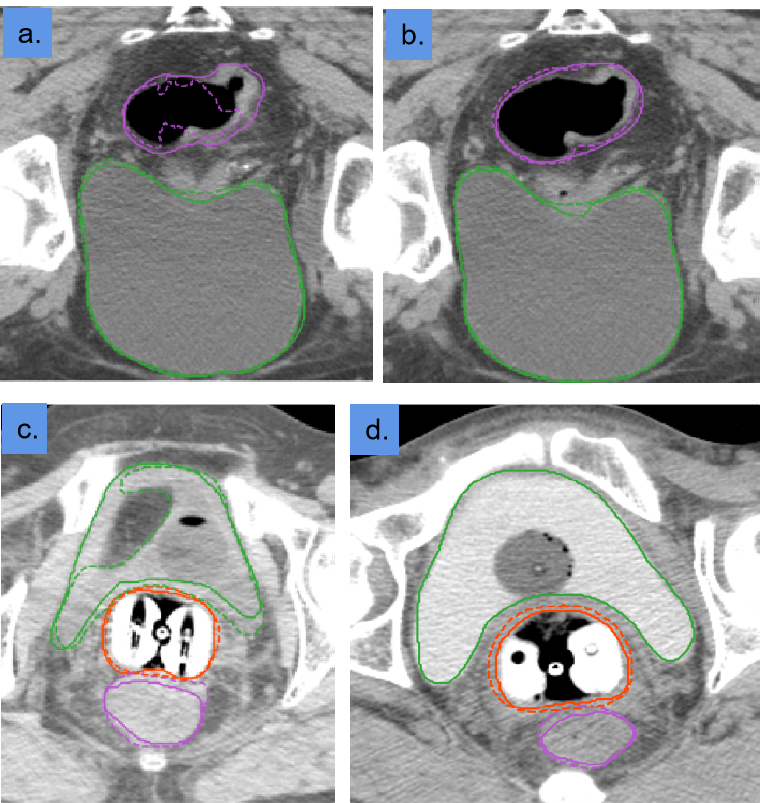


Figure 1. Visualization of segmentation demonstrated manual contouring (solid line) and auto-segmentation (dashed line): rectum (purple), bladder (green), and HRCTV (orange).

a&b. Minor segmentation errors were usually observed at the top slice of the rectum(c). The second slice has a good segmentation(d).

c. Major segmentation errors were noticed in cases where abnormal anatomy (e.g., large air bubbles in the bladder) exists.

d. Failed segmentation only occurred in one case with contrast enhanced agent resided in the bladder.

Table 2 Statistical analysis between different models.


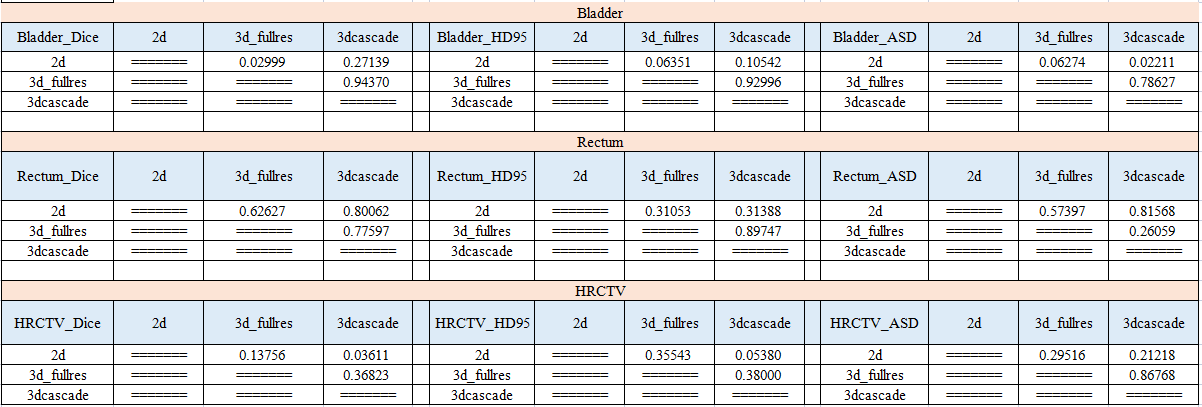


Table3 Training time for each architecture

| Training Time | |
| --- | --- |
| 2D | ~20h/fold |
| 3D-FullRes | ~65h/fold |
| 3D-LowRes | ~65h/fold |
| 3D-Cascade | ~70h/fold |
| Total | 5.2 Months |
